# Tissue Surface Adaptation and Clinical Performance of CAD-CAM Milled versus Conventional Implant-Assisted Mandibular Overdenture

**DOI:** 10.1155/2022/8220233

**Published:** 2022-06-16

**Authors:** Noha H. El-Shaheed, Hanadi A. Lamfon, Rabab. I. Salama, Amira Mohammed Gomaa Faramawy, Aisha Zakaria Hashem Mostafa

**Affiliations:** ^1^Prosthodontic Department, Faculty of Dentistry, Mansoura University, Mansoura, Egypt; ^2^Removable Prosthodontics, Faculty of Dentistry, Umm Al-Qura University, Makkah, Saudi Arabia; ^3^Pediatric Dentistry and Dental Public Health Department, Faculty of Dentistry, Mansoura University, Mansoura, Egypt

## Abstract

**Purpose:**

To evaluate the surface adaptation and maximal biting force of CAD-CAM milled mandibular overdenture (CAD-CAM MOD) compared to conventional compression mold mandibular overdenture (CC MOD).

**Materials and Methods:**

Ten completely edentulous subjects with persistent complaints of their complete mandibular dentures were received four dental implants in the anterior mandible. Three months after osseointegration, subjects were randomly received either conventional compression mold or CAD-CAM MOD in a crossover design. To assess tissue surface adaptation, the fitting surfaces of each denture base were scanned and placed on the reference master cast. Three and six months after each overdenture was inserted, clinical performance in the form of maximum biting force was evaluated.

**Results:**

The results of this study indicated that the tissue surface adaptation of the CAD-CAM MOD bases was significantly better than the conventional (compression mold technique) processed bases where (*P*=0.0001). Regarding clinical performance (maximum biting force), the CAD-CAM MOD exhibited better clinical performance (*P*=0.0001).

**Conclusions:**

In denture processing methods, the CAD-CAM overdenture delivered more precise adaption and clinical performance than the compression mold technique.

## 1. Introduction

Removable denture adaptation is well established to be the most important element in determining the quality of the prosthesis [1]. A well-adapted denture has higher primary wearing comfort and a lower incidence of traumatic ulcers [2]. Adequate denture tissue fit is also important for denture retention, stability, and support [3]. Denture retention has a significant impact on masticatory performance and speaking capacity, and thus on the subjects' quality of life [4]. As a result, one of the key goals of denture manufacture is to achieve maximum mucosal adaptability.

Removable dentures can be produced from a variety of materials and processing techniques. With the type of acrylic resin used and different construction procedures, denture adaption was considerably varied [5]. The compression molding technique, which has been used for decades, is the most extensively utilized processing technique. Although this method has several benefits, dentures might be distorted during processing [6, 7]. Dimensional changes can occur due to polymerization shrinkage and expansion, thermal shrinkage, water absorption, and internal stress release [8]. Denture base adaption to the underlying mucosa is reduced as a result of this deformation, resulting in decreased denture stability and retention. [3, 9].

By digital superimposition, the materials and techniques employed in 3D Printing produced diverging results and the lowest value for accuracy of the fitting surface of the denture foundation in comparison to the CAD/CAM milled base, injection, and compression mold techniques [10]. CAD-CAM produced dentures provide the best denture base adaptation [11–15]. Through the use of prepolymerized blocks of polymethyl methacrylate (PMMA), computer software, and a 5-axis milling machine, CAD-CAM produced dentures have emerged as a popular choice [16, 17], due to advancements in dental technology. In the dentistry field, CAD-CAM dentures have quickly acquired popularity [18, 19]. For both the maxillary and mandibular arches, CAD/CAM denture bases milled from PMMA blocks performed better adaptation than 3D printed, wax milled, and conventionally fabricated heat polymerized denture bases [9, 16].

In addition, the convenience of constructing additional dentures utilizing digitized recorded patient clinical data is one of the merits of CAD-CAM dentures compared to conventional dentures. As a result, if the denture is lost or broken, a replacement prosthesis can be made without the need for new clinical records [20, 21]. CAD-CAM dentures can be completed in two appointments rather than five using the traditional method, saving time for the dentist, technician, and patient [16, 17]. Furthermore, the least amount of distortion during CAD-CAM denture processing, which is critical for mucosal adaptation [14, 22].

The implant-assisted overdenture (IOD) is a well-known treatment option for overcoming the functional inefficiencies that come with traditional dentures [23, 24]. Despite the numerous advantages of implant-assisted prostheses, stress transfer and distribution due to occlusal pressures is expected to differ from that of a traditional complete denture [25]. Under functional forces, the implant acts as a fulcrum of rotating movement, causing concentration of great stresses in the attachment housing area and bone resorption on the edentulous area due to the greater resiliency of the mucosa covering the edentulous ridges compared to the rigid implant abutments. Furthermore, implant fracture, peri-implant bone loss, and subsequent implant failure are possible complications [26–29]. As a result, the goal of this study was to demonstrate the basic adaptability of overdenture.

Denture adaptation is one of the most critical factors determining the clinical performance of complete dentures. Well-fitted dentures provide comfort with fewer traumatic ulcers and greater chewing efficiency [30]. Many factors can influence chewing efficiency, including occlusal contact number, bite force, and masticatory muscle work to grind and break food [31]. The most accurate indicator of occlusal force is the maximal bite force (MBF) [32, 33].

Completely edentulous patients have a masticatory force that is 20%–40% that of healthy dentate people. As a result, complete denture wearers require up to seven times more chewing strokes than dentulous patients to masticate the food [26]. Two mandibular implants dramatically increase bit force and quality of life [34, 35]. Telescopic prostheses were similarly linked to increased MBF [36].

Researchers investigated the adaptation of maxillary [14, 22, 37] or mandibular [38] CAD-CAM complete dentures compared with conventional complete denture bases using superimposition analysis of scanned denture bases [14, 22, 38]. However, the research has not evaluated the tissue surface adaptation of implant-assisted mandibular overdenture bases fabricated by the CAD-CAM technique. Also, most of the studies carried out in CAD-CAM complete dentures are in vitro studies [14, 22, 38, 39], thus more clinical research is necessary to find out the situation. Studies assessing the tissue surface adaptation and clinical performance of CAD-CAM implant-assisted PMMA mandibular overdentures are required and hence emerged the aim of this study. Also, as previously stated, there is a correlation between denture base adaptation and chewing efficiency, which is influenced by biting force. There is a lack of clinical research of denture adaptation and its effect on the biting forces. As a result, the research initiative was created.

This study was aimed to evaluate the CAD-CAM processing technique of prepolymerized PMMA with respect to the denture base adaptation and maximum biting force of implant-assisted overdentures compared to conventional techniques for fabricating overdenture bases. The null hypothesis in this clinical trial is that there will be no differences in the above mentioned tissue adaptation, maximum biting force, and clinical performance with milled CAD-CAM or conventional compression mold manufactured implant-assisted overdenture.

## 2. Material and Methods

### 2.1. Study Design and Subjects' Criteria

**Figure 1:**
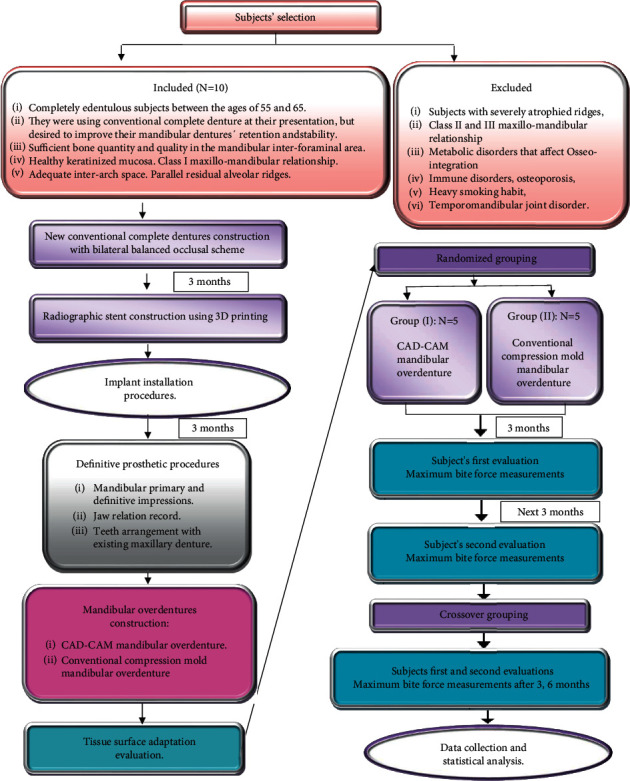
Subjects' selection, treatment procedures, and subjects' evaluation.

This prospective clinical study compared two mandibular complete overdentures constructed with two different techniques: milled CAD-CAM or conventional compression mold techniques, using a randomized crossover study design to assess mandibular overdenture base adaptation and maximum biting force. Subjects' selection, treatment procedures, and subjects' evaluation were summarized in Figure 1.

Ten completely edentulous subjects between the ages of 55 and 65 were chosen from the outpatient clinic of the Prosthodontic Department, Faculty of Dentistry, and Mansoura University. NCSS PASS Professional 2021 Software was used to compute the sample size for this investigation, which provided 80 percent power and a 0.05 alpha (*α*) for the paired *t*-test. The sample size was determined based on the program calculation and the previous research [40, 41].

Subjects included in their study were using conventional complete dentures at their presentation but desired to improve their mandibular dentures'sretention and stability. Included subjects were required to fulfill the following criteria: the subjects wore complete dentures, had sufficient bone quantity and quality in the mandibular interforaminal area required for standard implants of at least 10 mm length and 3.6 mm diameter provided by cone beam computed tomography, healthy keratinized mucosa, class I maxillo-mandibular relationship, adequate interarch space, and parallel residual alveolar ridges. The exclusion criteria included one or more of the following: subjects with severely atrophied ridges, class II and III maxillo-mandibular relationship, metabolic disorders that affect osseointegration such as uncontrolled diabetes mellitus, immune disorders, osteoporosis, heavy smoking habit, and temporomandibular joint disorder.

This study was approved by the ethical committee of the University of Mansoura, Faculty of Dentistry. The subjects signed an informed consent for participation in this study after they were informed about the full details of study procedures. Clinical study guidelines were followed.

### 2.2. Surgical and Prosthetic Procedures

New conventional complete dentures with a bilateral balanced occlusal scheme were fabricated for all subjects. Subjects were informed about the importance of wearing the dentures for 3 months before implant placement to improve neuromuscular adaptation.

As a radiographic stent, clear acrylic resin was employed with gutta percha markers. The individuals were scanned using cone beam computed tomography in accordance with the dual scan protocol [42]. Using 3D image planning software, the implants were virtually placed parallel to each other and perpendicular to the occlusal plane at the canines and lateral incisor locations (OnDemand 3D). Rapid prototyping was used to create a surgical stent with four sleeves positioned over possible implant locations (In2Guide).

Four implants (Dentium Co., Seoul, Korea) were inserted in the mandibular lateral incisors and canine regions bilaterally using a flabless technique and conventional loading protocol. Implants were inserted using the surgical stent which was fixed to the underlying bone using anchor pins. Osteotomy was done using the universal surgical kit supplied with the surgical stent. Implant fixtures were then inserted.

Healing abutments were screwed to the implants, and the mandibular dentures were relieved over the implant sites and relined using a soft liner material (promedica, Germany) to be used as a provisional denture.

**Figure 2:**
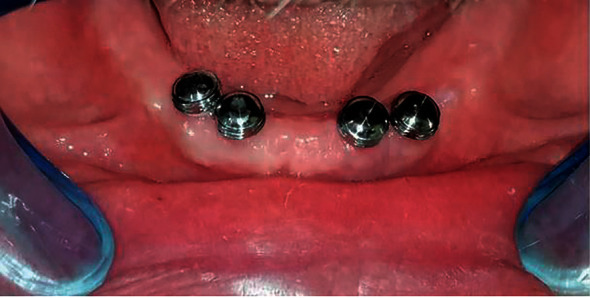
The processing caps and metal housings were inserted on the implant abutments.

Three months thereafter, the mandibular impression was started. The primary impression was recorded and poured to obtain the primary cast. A closed custom tray was constructed on the primary casts. The positioner attachments (Dentium, Co., Seoul, Korea) were screwed to the implants and the processing caps and metal housings were secured over the attachments (Figure 2). The definitive impression was recorded at abutment level using silicon impression material (Silaxil Light Body–LASCOD, Italy) in a border molded custom tray. The impression was poured with extra hard scannable dental stone (Kimberlit extra hard high-density die stone, Girona, Spain) after removing the processing caps and the metal housings from the impression to get the master cast. Maxillo-mandibular relations were recorded on mandibular conventional record blocks opposing the existing maxillary complete denture. Semianatomical acrylic teeth (Ruthinium acrylic teeth, Acry Rock Company, Italy) were set up in bilateral balanced occlusion and tried-in in the patient's mouth.

According to the mandibular denture base material and the processing technique, each subject was randomly given their mandibular overdentures in a crossover study design: five subjects were randomly given the CAD-CAM mandibular overdenture (CAD-CAM MOD) first, and the other five were given the conventional compression mold mandibular overdenture (CC MOD). After 3 and 6 months, maximum bite force was measured, and at 6 months, subjects who woreCAD-CAM MOD received CC MOD and vice versa. After another 3 and 6 months, the measurements were repeated. The sequence of delivering the mandibular overdentures was performed randomly to avoid impact of the prosthesis order on maximum bite force measurements. The randomization was performed using generated numbers in the Excel spread sheet by a person who was blinded to treatment groups.

**Figure 3:**
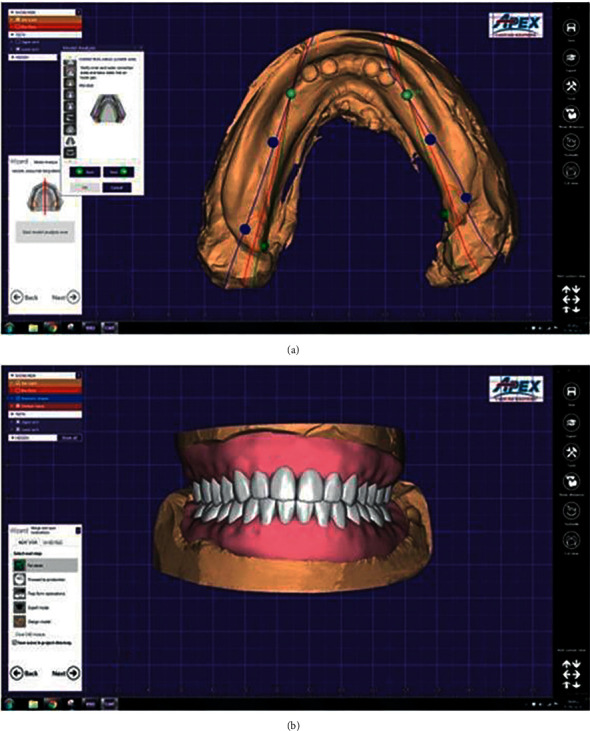
The virtual master cast (a) and virtual trial denture (b).

**Figure 4:**
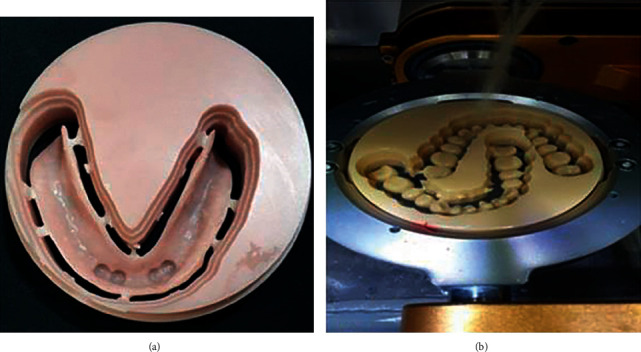
Denture base was fabricated from gingival-coloured prepolymerized PMMA blocks (a). The denture teeth were milled from tooth-colored prepolymerized PMMA blocks (b).

For the CAD-CAM MOD group, the mandibular master cast and record block were scanned both separately and while biting onto the recorded jaw relation, using an intraoral digital scanner (Medit I 500, Corea). The scanned data were saved as standard tessellation language (STL) file format. These data were uploaded into the design software (EXOCAD DentalCAD DB 2.2 valletta), where the undesirable scanned areas that outside the area of concern were detached and the files were merged using best-fit method. The virtual master cast that was used as a reference cast scan was created (Figure 3(a)). The anatomical landmarks were plotted on the reference cast and the denture base outline was determined. Finally, the mandibular denture base was designed virtually, and the teeth selection and setup were carried out (Figure 3(b)). Based on the virtually designed mandibular denture base, the final denture base was milled with a 5-axis milling machine with an accuracy of ±5 mm (MILL Box 2018 milling machine: ARUM 400, Corea) from gingival-colored prepolymerized PMMA blocks (PMMA Disc, bio HPP, Germany) (Figure 4(a)). Denture teeth were also milled from tooth-colored prepolymerized PMMA blocks (Figure 4(b)), finished, and then bonded into the milled base with a bonding agent (Visio Lign: Bredent).

For the CC MOD group, heat-cured polymethyl methacrylate resin (Major Prodotti Dentari S.P.A; Italy) was used [42]. The same master cast and record block that were previously scanned for CAD-CAM overdenture construction were used for conventional overdenture construction. Mandibular teeth were set up in a balanced occlusion with the existing maxillary denture. The waxed up denture was flasked using the compression mold technique. The acrylic resin polymer and monomer were thoroughly mixed according to the manufacturer's directions. The heat-cured acrylic resin was then packed and polymerized using the long curing cycle. Finishing and polishing were then done.

After the mandibular overdentures were constructed, the fitting tissue surface of each mandibular overdenture base was digitally scanned using the same intraoral digital scanner that was previously used. The obtained scanned 3D images were exported to a standard tessellation language (STL) file. The unwanted scanned points that were outside the area of interest (internal surface) were removed before superimposition.

### 2.3. Tissue Surface Adaptation Evaluation

Each STL file of the entire fitting surface of the scanned denture base was superimposed on the STL file of the reference scanned mandibular cast for each subject, with surface matching software [38, 43–45] (Geomagic verify; 3D system). Each scanned overdenture base was assessed for positive and negative average deviation values.

**Figure 5:**
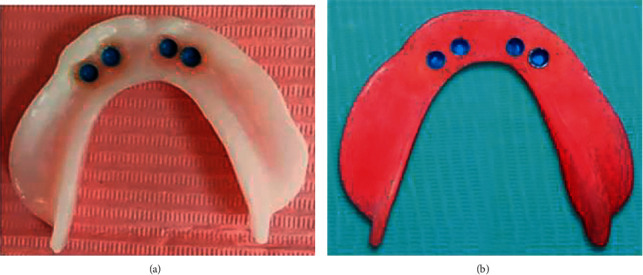
(a) Fitting surface of CAD-CAM milled mandibular overdentures with picked up attachments. (b) Fitting surface of conventional, compressed mold technique, constructed mandibular overdentures with picked up attachments.

At the insertion appointment, any adjustments that may be needed were carried out to ensure proper denture base fit, border extension, even occlusal contact and patient comfort. Self-cure acrylic resin was used to pick up the female housing attachment to the fitting surface of the mandibular overdenture bases (Figure 5(a), 5(b)). The white processing cap was removed and replaced by a blue one. The mandibular overdenture was delivered to each subject according to the included group.

### 2.4. Maximum Biting Force (MBF) Evaluation

**Figure 6:**
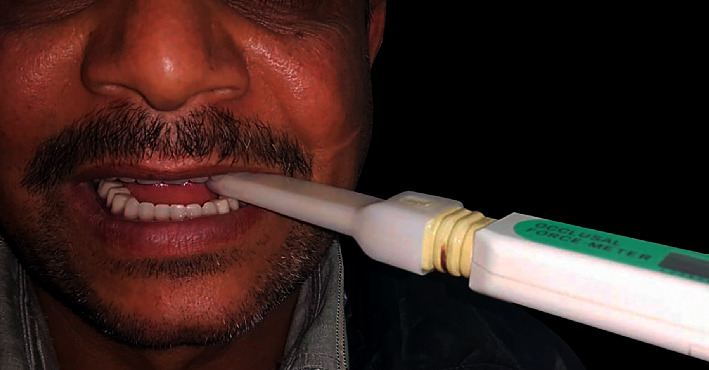
Patient exerts a maximum biting force on the bite force transducer.

A force transducer occlusal force meter (GM10, Nagano Keiki Co, Tokyo, Japan) was used to measure the subject's maximum biting force (MBF) (Figure 6). A digital hydraulic pressure gauge and a vinyl biting element with a plastic sheath make up this instrument. The pressure gauge's little digital screen displayed the maximum bite force values in Newtons (N). The participants sat in a dental chair in an upright position. The right side of the body was measured first, followed by the left side. Subjects were told to bite maximally for a few seconds while the transducer was placed horizontally between the occlusal surfaces in the first molar area. The measurement was done three times on each side, with a two-minute break in between. On the screen, the greatest bite force was recorded for each time. The highest of the three numbers was chosen. The mean of the left and right maximal bite force signals was used for statistical analysis.

### 2.5. Statistical Analysis

Collected data was analyzed using SPSS software version 20 (SPSS Inc., Chicago, IL, USA). The parametric data was displayed as mean (*M*) and standard deviation (±SD). Positive and negative average deviation means of the CAD-CAM MOD group and the CC MOD group were statistically compared using a paired *t*-test. Maximum bite force (MBF) within each group with different time measurements was compared using a paired *t*-test while comparison of MBF between the two groups at each evaluation time was performed using a *t*-test. *P* value was significant when ≤0.05 level.

## 3. Results

### 3.1. Tissue Surface Adaption of CAD-CAM MOD and CC MOD

The descriptive analysis (mean ± standard deviation) of positive and negative average deviations of CAD-CAM MOD and CC MOD is presented in Table 1.

The mean ± standard deviation of measured positive average deviation of the CC MOD was(0.099 mm ± 0.01), while the negative average deviation values were (−0.081 mm ± 0.009), respectively. The positive mean value of the CAD-CAM MOD was (0.034 mm ± 0.003) while the negative mean values were −0.055 mm ± 0,004.

Regarding positive average deviation values, the CAD-CAM MOD group was significantly lower than the CC MOD group (*P*=0.0001). Regarding negative average deviation values, the CC MOD group was significantly higher than those for the CAD-CAM MOD group (*P*=0.0001).So that, on the basis of analytical statistics, surface matching revealed that the CAD-CAM MOD group presented the higher values of tissue surface adaptation compared to the CC MOD group. The difference between the two tested groups was statistically significant.

### 3.2. Maximum Biting Force (MBF) by CAD-CAM PMMA Resin and Conventional Heat-Cured Processed Implant-Assisted Overdentures

The descriptive analysis (mean ± standard deviation) of MBF of CAD-CAM MOD and CC MOD is presented in Table 2.

CAD-CAM MOD group (Group I) presented the higher value of maximum biting force (208 ± 3.17), (225 ± 3.45) at 3 and 6 months, respectively, compared to CC MOD (Group II) (166 ± 4.19), (170 ± 4.30) at 3 and 6 months, respectively. The difference between the two tested groups was statistically significant (*P*=0.0001). The MBF was higher significantly at 6 months than at 3 months in both groups.

## 4. Discussion

The development of computer-aided technologies for constructing removable prosthesis is now progressing. More prospective clinical trials, however, are required to validate this method. The mechanical properties [46], trueness and tissue surface adaptation [40, 41], retentive quality [47], biocompatibility and microbial colonization [48], clinical outcomes and patient satisfaction [49, 50], and clinical complications and quality assessments [51, 52], of CAD-CAM complete dentures compared to conventional dentures were assessed.

However, there is a lack of evidence in the dental literature about implant-assisted overdenture base adaptation fabricated using the CAD-CAM technique and the resulting clinical performance when these dentures are supported by implants. The aim of this study was to evaluate implant-assisted overdenture base adaptation and, as a result, the clinical performance of these overdentures that are supported by implants when constructed using the CAD-CAM technique versus the conventional technique. In this study, the CAD-CAM-milled mandibular overdentures showed better fit and clinical performance compared to conventional compression molded overdentures so that the null hypothesis was rejected.

The unique manufacturing approach could be responsible for the superior fit of CAD-CAM produced dentures observed in this study. Because the denture base is milled from a fully prepolymerized resin puck that was polymerized at high temperature and pressure in a subtractive technique, as a result, volumetric variations associated with denture base processing are no longer an issue. As a result, when CAD-CAM manufactured dentures were compared to conventional dentures, the fitting surface of the CAD-CAM fabricated dentures revealed a higher similarity to the master cast surface, as previously explained [14, 22, 53].

This finding was in agreement with previously published articles comparing CAD-CAM processing techniques with pack and press [14] and reporting that CAD-CAM for denture fabrication process was more accurate and reproducible denture fabrication technique [14, 52].

On the other hand, conventionally fabricated dentures undergo distortion during processing [7], resulting in a negative impact on the denture base adaptation to the underlying mucosa [54, 55].

The significant increase in the maximum biting force while subjects wearing the CAD-CAM overdentures compared to conventional overdentures may be attributed to improvement in denture adaptation that in turn resulted in more patient comfort and more ability to bite without discomfort that was accompanied with the conventional denture [49]. This result was in accordance with Allahyari and Niakan who found better clinical retention and a reduced incidence of denture-related traumatic ulcers with CAD-CAM dentures and a[56] reduced number of postinsertion adjustment appointments [16].

Another explanation for biting force increasing after CAD-CAM denture wearing may be due to physical retention improvement of the prosthesis; hence, less effort was required from the muscles to retain or stabilize the prosthesis [47].

This is confirmed by Al Helal et al. [47] who reported that retention offered by milled complete denture bases from prepolymerized polymethyl methacrylate resin was significantly higher than conventional heat polymerized denture bases.

On the contrary, the conventionally fabricated dentures show a combination of some areas of more adaptation than others, resulting in some mucosal impingement in some areas which results in sore spots and patient discomfort, and others that were out of contact creating compromised retention. This most likely increases the clinician's chair time because of additional adjustments [56]. This may explain why conventional dentures have reduced bite force.

The significant increase in the biting force for conventional fabricated dentures and CAD-CAM fabricated dentures with time from three to six months may be owing to the progressive experience establishment in addition to increased denture base adaptation by time as confirmed by many earlier studies [57, 58].

This study has some strengths. The crossover design utilized in this study was aimed to reduce human variability and to standardize the tested prosthetic appliances for clinical performance evaluation, using two identical overdentures for each subject.

The clinical performance evaluation was initiated three months after overdenture insertion to provide adequate time for proper neuromuscular accommodation to the new prosthesis as previously reported [59].

Another important strength in this study was the use of laser scanners for assessing the dimensional changes that occur during denture production. Many methods have been developed previously, but the recently introduced laser scanners have been proven to be a reliable means of determining denture base adaption. This technology is used for measurements by superimposing and analyzing scanned information using cutting-edge computer software [44, 60].

Also, in this study, instead of using geometric reference points for surface matching [15], the entire fitting surfaces of the master cast and constructed denture bases were evaluated [23, 26, 27]. Thus all possible deviations over the entire fitting surfaces of the denture bases were recorded.

However, one inherent limitation in this study was the relatively small number of participants. However, because a crossover study was utilized, a small sample size can be used compared to parallel group studies [61].

Another limitation in this study was that the effect of the previous prosthesis type on the MBF of the current prosthesis was not measured. A rest period may be needed before making the crossover. This factor should be considered in future research.

Further long-term clinical trials with increased sample size are needed to evaluate further clinical aspects of CAD-CAM milled overdentures.

## 5. Conclusion

Within the limitations of the current study, it can be stated that restoring the edentulous mandible with CAD-CAM constructed implant-assisted overdentures increases tissue surface adaption and maximal biting force when compared to conventionally fabricated acrylic resin overdentures.

## Figures and Tables

**Table 1:** Descriptive analysis of measured surface deviations between scanned master casts and scanned overdenture bases fabricated by conventional and CAD/CAM milled techniques.

	Positive average values			Negative average values		
	CC MOD	CAD-CAM MOD	t value (*P* value)	CC MOD	CAD-CAM MOD	t value (*P* value)
M	0.099	0.034	20.77 (0.0001)^*∗*^	−0.081	−0.055	10.22 (0.0001)^*∗*^
±SD	0.01	0.003		0.009	0.004	

^*∗*^Statistically significant difference. M, mean; SD, standard deviation; CC MOD, conventional compression mold overdenture; CAD-CAM MOD:CAD-CAM mandibular overdenture; (-) average, negative average values; (+) average, positive average values.

**Table 2:** Descriptive analysis of maximum biting force (MBF) values and*P* values of conventional PMMA and CAD/CAM PMMA implant overdenture prostheses along the various follow-up periods.

Time of measurement	Processing technique (denture type)			
	CC MOD	CAD-CAM MOD	t value	*P* value
	M ± SD	M ± SD		
3 months	166 ± 4.19	208 ± 3.17	30.96	(0.0001)^*∗*^
6 months	170 ± 4.30	225 ± 3.45	31.54	(0.0001)^*∗*^
t value	2.16	11.47		
*P* value	(0.05)	(0.0001)^*∗*^		

^*∗*^Statistically significant difference. M,mean; SD, standard deviation; CC MOD, conventional compression mold overdenture CAD-CAM MOD:CAD-CAM mandibular overdenture.

## Data Availability

Data are available from the corresponding author upon reasonable request.
